# Determining host factors contributing to the reactivation of JC virus in kidney transplant recipients

**DOI:** 10.1186/s12985-022-01843-w

**Published:** 2022-08-08

**Authors:** Sajedeh Keykhosravi, Masoud Khosravi, Mohammad Shenagari, Elham Hasan-alizadeh, Mehrdad Mosadegh, Narjes Noori Goodarzi, Ali Monfared, Babak Ashrafkhani, Tolou Hasandokht

**Affiliations:** 1 Department of Microbiology, Faculty of Medicine, Guilan University of Medical Sciences, Rasht, Iran; 2 Urology Research Center, Guilan University of Medical Sciences, Rasht, Iran; 3 Organ Transplant Research Center, Guilan University of Medical Sciences, Rasht, Iran; 4 Faculty of Medicine, Guilan University of Medical Sciences, Rasht, Iran; 5 Department of Pathobiology, School of Public Health, Tehran University of Medical Sciences, Tehran, Iran; 6 Department of Community Medicine, Faculty of Medicine, Guilan University of Medical Sciences, Rasht, Iran

**Keywords:** JC virus, Renal transplantation, Graft rejection, Reactivation, Risk factors

## Abstract

**Background and aims:**

The John Cunningham virus (JCV) is the established etiological agent of the polyomavirus-associated nephropathy among renal transplant recipients. In the present study, we aimed to determine the probable predictive factors leading to JCV replication in renal transplant patients.

**Material and methods:**

Urine and plasma samples were collected from a total of 120 consecutive renal‐transplanted patients without preliminary screening from Jan 2018 to Mar 2019. After DNA extraction, the simultaneous detection and quantification of JCV and BK polyomavirus (BKV) were conducted using a Real-time quantitative PCR method. Moreover, statistical analyses were performed using the statistical software packages, SPSS version 21.

**Results:**

The prevalence of JCV viruria and viremia among renal transplant recipients were 26 (21.67%) and 20 (16.67%), respectively. A significant association was observed between the JCV and two risk factors, diabetes mellitus (*P* = 0.002) and renal stones (*P* = 0.015). The prevalence of JCV viremia among recipients who were grafted near time to sampling was significantly higher (*P* = 0.02). There was a statistically significant coexistence between BK and JC viruses among our patients (*P* = 0.029). The frequency of JCV viruria in males was reported almost three times more than in females (*P* = 0.005). The JCV shedding in urine was significantly associated with the tropical steroids like prednisolone acetate, which have been the standard regimen (*P* = 0.039). Multivariable analysis revealed duration of post-transplantation (OR, 0.89; *P* = 0.038), diabetes mellitus (OR, 1.85; *P* = 0.034), and renal stone (OR 1.10; *P* = 0.04) as independent risk factors associated with JCV viremia post-renal transplantation.

**Conclusion:**

It seems that the discovery of potential risk factors, including immunological and non-immunological elements, may offer a possible preventive or therapeutic approach in the JCV disease episodes. The results of this study may also help clarify the probable clinical risk factors involving in progressive multifocal leukoencephalopathy development.

## Introduction

The John Cunningham virus (JCV) is an ubiquitous human polyomavirus that was first discovered in 1971 from patients with progressive multifocal leukoencephalopathy (PML) [1]. Until now, 14 polyomaviruses with human hosts have been identified. Structural and antigenic studies have revealed that JCV is closely related to BKV, which was coincidently discovered and associated with nephropathy and graft rejection in renal transplant recipients [2]. Recent seroepidemiological data indicate that the JCV asymptomatically infects up to 80% of the world's population [3]. Thereafter, it establishes a latent infection in the renal, hematopoietic progenitor cells, peripheral blood B lymphocytes, and tonsillar stromal cells and reactivates itself from latency under immunocompromised conditions [4]. In healthy individuals, the JCV can reactivate and shed in urine without any functional impairment in renal function [5].

Nevertheless, in immunocompromised renal allograft recipients, JCV can bring about nephropathy and/or PML [6]. PML occurs mainly in acquired immunodeficiency syndrome (AIDS) patients and less frequently after solid organ or allogeneic hematopoietic stem cell transplantations and in Multiple Sclerosis or Crohn's disease patients treated with immunomodulatory drugs [7]. Furthermore, PML also occurs rarely in renal transplant patients [8]. The prevalence and quantification of the BKV and JCV urinary loads could be higher in immunosuppressed compared to non-immunosuppressed individuals; however, this report remains controversial surrounding JCV [9]. Like the BKV, the JCV has also been noted to be a cause of urethral stenosis [10]. There have been some reports regarding JCV-associated nephropathy in BKV negative renal transplant recipients. Thus, monitoring of JCV infection, especially during the first 24 months after transplantation, is recommended by some nephrologists. Despite the high incidence of reactivation of the JC virus in recipients of renal transplantation, a small fraction of patients eventually show JC virus-associated nephropathy, which may lead to rejection of the transplant [11]. As with BKV nephropathy, serologic studies support reactivation of endogenous JCV rather than a primary infection [12, 13]. Thus, some studies have been conducted to assess the probable risk factors leading to polyomavirus reactivation and the resulted nephropathy [14–16]. However, JC polyomavirus associated nephropathy (PyVAN) is a unique clinical entity that needs to be differentiated from BK PyVAN, and there was no comprehensive study examining the risk factors proceeding JCV replication and nephropathy in renal transplant patients. Regarding the extent of renal transplantation worldwide, it is mandatory to identify underlying causes of nephropathy and graft rejection to propound algorithms for ensuing nephropathy as far as possible. The relationship between JCV replication and immunosuppression is less well defined than that with the BK virus. This is the first study to evaluate the probable risk factors contributing to JC virus replication post-transplantation. The present study aims to assess the reactivation of JCV from a latent or non-productive state to a productive infection in renal cells that can lead to graft failure in renal transplant recipients.

## Material and methods

### Subjects

In the present descriptive cross-sectional study, we collected urine and plasma samples from 120 consecutive renal transplant recipients by referring them to the Molecular Diagnostic Center, Guilan University of Medical Sciences, between January 2018 and March 2019. No pre-transplant JC virus status data were available because most individuals are infected with this virus as an opportunistic viral infection during their life span. Among our patients, there was no prophylaxis, intervention, and treatment for PyVAN. Besides, no preliminary screening was used to allow or prevent patient enrollment into the study, and all samples were processed. Each patient was screened only once. Data regarding demographics, underlying diseases, and immunosuppressive regimens in the studied patients were collected.

### Sampling and quantitative real-time PCR

JCV and BKV replication was employed as a detectable viral genome in plasma and urine samples of renal transplant recipients. One hundred twenty paired plasma, and urine specimens were stored at − 80 °C and used to quantify and simultaneously detect the JCV and BKV. The urine samples were centrifuged (1000*g* for 20 min) before DNA purification to assure the sedimentation of the cells containing the virus. DNA was extracted from 0.2 ml of urine and plasma using the QIAamp DNA mini kit (Qiagen, Hilden, Germany) according to the manufacturers' instructions. Simultaneous detection and quantification of JCV and BKV were performed in a StepOne Plus™ instrument (Applied Biosystems, Foster City, CA, USA) using the GeneProof™ Real-time PCR kits (Vídeňská, Czech Republic). The cycling steps were set as follows: initial denaturation at 95 °C for 10 min, followed by 45 cycles of 95 °C for 5 s and 60 °C for 40 s and 72 °C for 20 s.

### Statistical analysis

The probable predictive factors for JC virus replication were analyzed using the statistical software packages, SPSS version 21. The Shapiro–Wilk test was used to assess whether data were distributed normally. The result was reported as mean (standard deviation) or frequency (percentage) for numerical or qualitative data. Baseline characteristics were compared using a t-test or chi-square test. We used the chi-square test to compare the frequency of qualitative variables according to the presence of JCV in plasmas or urines. Also, an independent t-test was used to assess the numerical variables in groups with and without the JCV. The association between JC and BK viruses was assessed by the chi-square test. A 2-tailed *P* < 0.05 was regarded as statistically significant. The univariate logistic regression (ENTER method) and multivariate analysis using the BACKWARD model were performed to assess the effect of factors with a potential impact on JCV replication development.

## Results

### Transplant variables and JCV occurrence

As shown in Table 1, the mean age of the enrolled participants was 48.08 ± 15.26 years. The mean sampling time after transplantation was 9.91 ± 6.38 years. There were 65 (54.2%) males and 55 (45.8%) females. Renal stones with a frequency of 12.5% and high blood pressure with a frequency of 10% were the most common underlying diseases in the studied population. The most prescribed immunosuppressive medications were Prednisolone Acetate (93.3%) and Mycophenolate Mofetil (67.5%).

**Table 1:** Baseline characteristics of study population

Characteristics	Mean (SD)
Age, mean (SD)	48.08 (15.26)
Duration of transplant, year, mean (SD)	9.91 (6.38)
	n (%)
Sex	
Female	55 (45.8)
Male	65 (54.2)
DM	7 (5.8)
High blood pressure	12 (10)
Glomerulonephritis	8 (6.7)
Alport syndrome	5 (4.2)
Urinary reflux	6 (5)
Nephrotic syndrome	6 (5)
Polycystic renal	10 (8.3)
Renal stone	15 (12.5)
Sirolimus	4 (3.3)
Mycophenolate mofetil	81 (67.5)
Cyclosporine	48 (40.0)
Tacrolimus	17 (14.2)
Mycophenolic acid	15 (12.5)
Azathioprine	2 (1.7)
Prednisolone Acetate	112 (93.3)

We used a Real-time PCR test to detect and quantify the JCV among renal transplant recipients. The prevalence of JCV viruria and viremia was 26 (21.67%) and 20 (16.67%), respectively. The frequency of JCV viruria in males (30.7%) was reported almost three times more than that of females (10.9%), which was statistically significant (*P* = 0.005). The frequency of JCV viremia in diabetic patients (57.1%) was significantly higher than that of the non-diabetic group (*P* = 0.002). There was also a significant relationship between JCV viremia and renal stones' history (*P* = 0.01). Diabetes mellitus could act as a significant risk factor for the shedding of the JCV in blood. The prevalence of the JCV among recipients who were grafted in near time to sampling had a higher incidence in comparison to patients with elapsed time post-transplantation (*P* = 0.02). No significant relation was seen between the reactivation of the JCV and other factors such as age, acute rejection, and mean of Creatinine Level (CR) and Glomerular Filtration Rate (GFR) (*P* > 0.05) (Table 2).

**Table 2:** Demographic and clinical characteristics of renal transplant recipients and incidence of JC virus

	JC viruria		*P* value	JC viremia		*P* value
	JC positive (n = 26)	JC negative (n = 94)		JC positive (n = 20)	JC negative (n = 100)	
Age, mean (SD)	49.07 (15.01)	47.78 (15.40)	0.701	47.37 (14.87)	48.21**(**15.40)	0.827*
Sex						
Female	6 (10.9)	49 (89.1)	**0.005**	8 (14.5)	47 (85.5)	0.722^#^
Male	20 (30.7)	45 (69.2)		12 (18.4)	53 (81.5)	
Immunosuppression regimen						
Sirolimus	1 (25.0)	3 (75.0)	0.903	0 (0.0)	4 (100.0)	0.236^#^
Mycophenolate Mofetil	19 (23.5)	62 (76.5)	0.718	11 (13.6)	70 (86.4)	0.330^#^
Cyclosporine	12 (25.0)	36 (75.0)	0.592	7 (14.6)	41 (85.4)	0.759^#^
Tacrolimus	3 (17.6)	14 (82.4)	0.605	3 (17.6)	14 (82.4)	0.825^#^
Mycophenolic acid	3 (20.0)	12 (80.0)	0.804	2 (13.3)	13 (86.7)	0.777^#^
Azathioprine	0 (0.0)	2 (100.0)	0.310	0 (0.0)	2 (100.0)	0.404^#^
Prednisolone acetate	27 (24.1)	85 (75.9)	**0.039**	20 (17.9)	92 (82.0)	0.091^#^
CR, mean (SD)	1.66 (2.10)	1.41 (0.68)	0.329	1.93 (2.55)	1.38 (0.611)	0.365*
GFR, mean (SD)	63.30 (18.34)	69.19 (27.97)	0.202	70.16 (26.16)	67.44 (26.27)	0.679*
Duration post-transplantation	8 (4.97)	10.47 (6.65)	**0.041**	7.21 (4.96)	10.42 (6.50)	0.020*
Diseases						
Diabetes	3 (42.9)	4 (57.1)	0.216	4 (57.1)	3 (42.9)	**0.002**^#^
High blood pressure	5 (41.7)	7 (58.3)	0.116	1 (8.3)	11 (91.7)	0.420^#^
Glomerulonephritis	2 (25.0)	6 (75.0)	0.862	0 (0.0)	8 (100.0)	0.091^#^
Alport syndrome	2 (40.0)	3 (60.0)	0.370	0 (0.0)	5 (100.0)	0.184^#^
Nephrotic syndrome	26 (23.0)	87 (77.0)	0.722	1 (16.7)	5 (83.3)	0.840^#^
Renal stone	1 (33.3)	2 (66.7)	0.664	2 (66.7)	1 (33.3)	**0.015**^#^
Polycystic renal	(20.0)	8 (80.0)	0.841	0 (0.0)	10 (100.0)	0.507^#^
Urinary reflux	2 (33.3)	4 (66.7)	0.533	1 (16.7)	5 (83.3)	0.955^#^
BK virus co-existence	4 (15.4%)	11 (11.7%)	0.415	6 (31.6%)	11 (10.9%)	**0.029**^#^

Significant *P* values (< 0.05) are in bold

*Independent *t* test, ^#^chi square

### JCV replication and immunosuppressive regimens

Among the 120 recipients, the prescribed immunosuppressive medications were as follows: Prednisolone acetate for 112, Mycophenolate mofetil for 81, Cyclosporine for 48, Tacrolimus for 17, Mycophenolic acid for 15, Sirolimus for 4, Azathioprine for two patients. We examined the difference in immunosuppressant medications between JCV positive and negative groups. Based on our findings, treatment with Cyclosporine, Mycophenolic acid, Tacrolimus, Sirolimus, and Azathioprine did not affect the virus replication (*P* > 0.05). Patients who received Prednisolone acetate had a higher chance of shedding the JCV in the urine. Twenty-four percent of those taking Prednisolone acetate were JCV positive, and all those who did not use Prednisolone acetate were negative for the virus (*P* = 0.03) (Table 2).

### JC virus and BK virus coexistence

All 120 renal transplant recipients were analyzed simultaneously for the BKV and JCV replication, using the Real-time PCR assay. The association between JC and BK viruses was assessed by the chi-square test. Based on our observation, a significant association between BKV and JCV viremia was seen among our patients. BKV replication in renal transplant recipients facilitates JCV shedding or vice versa (*P* = 0.029) (Table 2).

### JC viral load and demographic features

The mean JC viral load in urine was 11 × 10^6^ ± 55 × 10^6^ copies/mL; range, 28 to 543 × 10^6^ copies/mL. Moreover, The mean JC viral load in plasma was and 1.8 × 10^6^ ± 7.4 × 10^6^ copies/mL; range, 28 to 543 × 10^6^ copies/mL. The mean load of the urinary JCV was ten times more than that of the plasma viral load. High-level JCV viruria was defined as JCV viruria > 10^7^ copies/mL, and high-level JCV viremia was defined as plasma JCV replication > 10^4^ copies/mL. Furthermore, we examined the probable effects of demographic elements of renal transplant recipients on the JC viral load by determining a cutoff of ≥ 4 log10/mL for plasma and ≥ 7 log10/mL for urine. Although the agreed cutoff is not available for JC PyVAN, we have considered the cutoff of ≥ 4 log10/mL for plasma and ≥ 7 log10/mL for urine based on the recommended and consensus cutoffs for BK PyVAN. As noted in Table 3, urinary reflux can increase the plasma JC viral load (*P* = 0.019). Besides, Tacrolimus medication has a statistically significant effect on increasing the JC viral load in plasma (*P* = 0.019). Based on our findings, the urinary JC viral load is likely to increase over the time of post-transplantation (*P* = 0.002). However, the lack of statistical significance about other elements might be due to a limited number of JCV-positive individuals.

**Table 3:** The table shows the different element and their impact on JCPyV viral load

	JC viral load in plasma		*P* value	JC viral load in urine		*P* value
	> 4 log10/ml (n = 5)	< 4 log10/ml (n = 15)		> 7 log10/ml (n = 2)	< 7 log10/ml (n = 24)	
Age, mean (SD)	45.60 (10.38)	48.33 (15.93)	0.726	38.50 (17.67)	49.79 (15.17)	0.325
Sex						
Female	3 (60.0%)	5 (33.3%)	0.292	1 (50.0%)	5 (20.8%)	0.347
Male	2 (40.0%)	10 (66.7%)		1 (50.0%)	19 (79.2%)	
Immunosuppression regimen						
Sirolimus	5 (100.0%)	15 (100.0%)		0 (0.0%)	1 (4.2%)	0.768
Mycophenolate Mofetil	3 (60.0%)	9 (60.0%)	1.000	1 (50.0%)	17 (70.8%)	0.540
Cyclosporine	1 (20.0%)	6 (40.0%)	0.417	1 (50.0%)	11 (45.8%)	0.910
Tacrolimus	2 (40.0%)	2 (13.3%)	0.197	1 (50.0%)	1 (4.3%)	**0.019**
Mycophenolic acid	1 (6.7%)	1 (20.0%)	0.389	0 (0.0%)	3 (12.5%)	0.595
Azathioprine	5 (100.0%)	15 (100.0%)		2 (100.0%)	24 (100.0%)	
Prednisolone acetate	5 (100.0%)	15 (100.0%)		2 (100.0%)	24 (100.0%)	
CR, mean (SD)	3.32 (4.86)	1.39 (0.851)	0.424	1.05 (0.21)	1.48 (0.16)	0.67
GFR, mean (SD)	78 (23.88)	69.40 (27.39)	0.540	73 (1.41)	67.78 (26.3)	0.359
Duration post-transplantation	8.80 (8.98)	7.66 (4.80)	**0.002**	7.5 (2.12)	7.45 (4.35)	0.990
Diseases						
Diabetes	0 (0.0%)	4 (26.7%)	0.197	0 (0.0%)	3 (12.5%)	0.595
High blood pressure	0 (0.0%)	1 (6.7%)	0.554	0 (0.0%)	5 (20.8%)	0.473
Glomerulonephritis	5 (100.0%)	15 (100.0%)		0 (0.0%)	2 (8.3%)	0.671
Alport syndrome	5 (100.0%)	15 (100.0%)		0 (0.0%)	2 (8.3%)	0.671
Nephrotic syndrome	0 (0.0%)	1 (6.7%)	0.554	1 (50.0%)	1 (4.2%)	0.019
Renal stone	0 (0.0%)	2 (13.3%)	0.389	0 (0.0%)	1 (4.2%)	0.768
Polycystic renal		5 (100.0%)	15 (100.0%)	0 (0.0%)	2 (8.3%)	0.671
Urinary reflux	0 (0.0%)	1 (6.7%)	0.554	1 (50.0%)	1 (4.2%)	**0.019**

The cutoffs of ≥ 7 log10/ml and ≥ 4 log10 were defined for urinary and plasma levels of JC virus, respectively

Significant *P* values (< 0.05) are in bold

### Multiple analyses for virus replication

In the multiple analyses, a logistic regression model was used to determine the factors associated with the frequency of JCV replication. In the final model among the studied variables, the time of post-transplantation was considered as a predictor of the frequency of the JCV for both viruria (OR 0.92, *P* = 0.05, 95% CI 0.8–0.98) and viremia (OR 0.89, *P* = 0.038, 95% CI 0.8–0.9). The prevalence of the JCV among recipients who were grafted in near time to sampling was higher in comparison to patients with elapsed time post-transplantation. Gender was also considered a predictor in the final model, so males had a higher chance of having JCV viruria than females. (OR 0.3, *P* = 0.02, 95% CI 0.1–0.8). Furthermore, diabetes mellitus (OR 1.85, *P* = 0.034, 95% CI 0.5–2.31) and renal stone (OR 1.10, *P* = 0.04, 95% CI 0.9–1.28) were found as predictor factors for JCV viremia. (Table 4).

**Table 4:** The table shows the statistically significant variables affecting the JC virus replication analyzed by multivariate regression

	ODDS ratio	95% CL for odds ratio		*P* value
		Lower limit	Upper limit	
Viremia				
Duration post-transplantation	0.89	0.8	0.9	**0.038**
Diabetes	1.85	0.5	2.31	**0.034**
Renal stone	1.10	0.9	1.28	**0.04**
Viruria				
Duration post-transplantation	0.92	0.8	0.98	**0.05**
Sex (female)	0.3	0.1	0.8	**0.02**

Significant *P* values (< 0.05) are in bold

## Discussion

Since its discovery in 1971, the most comprehensive studies about JCV have often been flexed toward the ability of this ubiquitous agent to cause a severe illness, PML, in immunocompromised individuals [17–20] Although the role of the JCV in developing nephropathy post-renal transplantation was confirmed, the assessments of probable risk factors associated with JCV reactivation were likewise more inclined toward the development of PML [21–24]. There is now a hypothesis that four essential conditions related to the development of the JCV-associated PML are as follows: (a) a significant immunosuppression (b) recombination in viral promoter allowing efficient transcription and replication in permissive cells (c) presence of cellular host factors to interact with the viral promoter in permissive cells d) and the ability of the virus to cross the blood–brain barrier. Since JCV reactivation often occurs in immunocompromised patients, it has been proposed that the host immune system (especially cell-mediated immunity) plays an incredible role in controlling the JCV by preventing its reactivation and PML development [25]. Although the exact immunological and non-immunological mechanism of JCV reactivation is not clear, detailed studies have been conducted to assess some intracellular elements' impact on JCV regulation. Serine/Arginine-Rich Splicing Factor 1 (SRSF1) possesses a strong negative regulatory effect of JCV replication [26]. However, the JCV Large T antigen counteracts the antiviral effects of SRSF1 protein [27]. Besides, the regulation of JCV transcription is influenced by some cellular trans-activator elements, including nuclear factor 1x (NF-1), NF-κB, c-Jun, Y-box binding protein 1, and Tst-1. NF-1X is overexpressed in cells permissive for JCV replication, including B-lymphocytes, glial cells, and tonsillar stromal cells [28, 29]. The binding of NF-1X to the JCV promoter region increases the expression of VP1, which is an indicator of increased expression of viral genes [28]. The human immunodeficiency virus (HIV) Tat protein has also been shown to interact with JCV, primarily through increasing the transcription of both early and late regions of JCV, and increases JCV propagation when infected cells express Tat [30]. Although most of the abovementioned studies have been focused on elucidating the molecular and immunological mechanisms involved in virus reactivation leading to PML development, similar mechanisms are likely to be involved in virus reactivation post-renal transplantation. Despite the JCV's confirmed role in developing nephropathy, very few studies have been published regarding the replication of JCV in transplant patients. JC PyVAN is a unique clinical entity that needs to be differentiated from BK PyVAN [31–34]. It seems that the BK and JC viruses have different mechanisms of reactivation and shedding. Although the role of immunosuppression in the onset of virus replication and shedding in urine has been controversial, immunosuppression is a prerequisite for JCV viremia [25]. The same event occurs about BKV replication and shedding in plasma as well [35]. A significant correlation between JCV viremia and BKV viremia was seen among our participants proposing mutual support and functional interplay between JC and BK viruses by applying probable molecular interaction. Since these two viruses share overlapping latency sites, the proliferation of one may stimulate the other's reactivation. The relevance of the BKV and JCV regulatory elements on the triggering of replication of polyomaviruses and cellular regulatory factors merits further study. Even though, Cheng and his colleagues pointed to the inhibitory interactions between BK and JC viruses on each other’s urinary shedding [36], the present study confirmed the synergistic interplay between them to increase the chance of viral replication and shedding in plasma.

Evidence and observations indicate the high prevalence of BK and JC viruses in Asian countries [37]. Korean population has the highest frequency of BKV (66.7%) [38], while the highest occurrence of JCV is reported from Taiwan (88%) [37]. The prevalence of JCV in previous studies from Iran ranges between 1.6 and 38% [39–41]; this variability might be due to different sampling methods and different populations.

In our study, the prevalence of JCV viruria and viremia among renal transplant recipients were 26 (21.67%) and 20 (16.67%), respectively. Some studies have mentioned that the prevalence of asymptomatic viruria is not increased in renal transplant recipients. Nonetheless, previous studies have been reported JCV genomic DNA in renal biopsy tissue and/or urine within a range of 3.4% and 46% of renal transplanted patients, while JCV viremia ranged from 0 to 25% [42–46]. A long-term prospective follow-up study was conducted in France, and JCV was detected in only 31 blood samples out of 1487 collected [47]. Since a low level of JCV replication and shedding is common in immunocompetent individuals, the association between the JCV viruria and transplantation has not been proved yet. Our results indicated that the median urinary JC viral load among our participants was 11 × 10^6^ ± 55 × 10^6^ copies/mL; range, 543 to 28 × 10^6^ copies/mL. The median JC viral load in plasmas was 1.8 × 10^6^ ± 7.4 × 10^6^ copies/mL; range, 543 to 28 × 10^6^ copies/mL. The median quantity of urinary JC viral load virus was approximately 10 times more than that of the plasma viral load. On the contrary, the low level of JCV viremia has been reported in previous studies in patients, both shedding large amounts of JCV in urine and with parenchymal involvement. The most recent studies also reported an extensive range of JC viral loads, from 2.0 × 10^3^ copies/mL to 1 × 10^7^ copies/mL [11, 42, 44, 48, 49]. In a cohort study involving 103 renal transplant recipients, the JCV was detected in 14.2% of subjects with a mean viral load of 2 × 10^3^ copies/mL [42]. Other studies demonstrated JCV load was markedly increased in transplant patients compared to healthy individuals, confirming the association between immune function and viral levels [50].

Contrary to some studies that have mentioned no effects of immunosuppressive drugs on the JCV reactivation, we demonstrated that Prednisolone Acetate medication could act as a significant risk factor for developing the JCV viruria. Besides, the Tacrolimus regimen also boosts the plasma level of JC viral load in renal transplant recipients. Nevertheless, the impact of Tacrolimus medication on JC viral load might not be virtual and, perhaps it is due to the low number of patients treated with this drug in our study. The correlation between sex and replication of polyomaviruses has been mentioned by some scientists [51]. In this study, we also showed that males had a higher chance of JCV viruria than females. In the current study, transplantation's mean duration was significantly shorter in groups with JCV viremia and viruria. Thus, the JCV among recipients who were grafted in near time to sampling had a higher incidence in comparison to patients with elapsed time post-transplantation. Inversely, the JC viral load in plasma was likely to increase over the time of post-transplantation. The plasma level of the JCV could be increased by urinary reflux as well.

## Conclusion

In our study, the frequency of JCV viremia in diabetic patients was significantly higher than that of non-diabetic groups. Also, there was a significant relationship between JCV viremia and history of renal stones.

Since there is no effective anti-viral agent for JCV nephropathy and the reduction of immunosuppression has a controversial impact on the clinical course, it seems that the discovery of probable risk factors including immunological and non-immunological elements may offer possible preventive or therapeutic approach in the JCV diseases episodes. The results of this study may also help clarify the probable clinical risk factors involving in PML development.

## Data Availability

All data generated or analyzed during this study are included in this published article.

## Abbreviations

GFR: Glomerular filtration rate
Cr: Creatinine level
CI: Confidence interval
OR: Odds ratio
DM: Diabetes mellitus
SD: Standard deviation
BKPyVAN: BK polyomavirus associated nephropathy
PML: Progressive multifocal leukoencephalopathy
AIDS: Acquired immunodeficiency syndrome
BKPyV: BK polyomavirus
JCPyV: JC polyomavirus
